# Platelet-Rich Plasma for Knee Osteoarthritis: A Comprehensive Narrative Review of the Mechanisms, Preparation Protocols, and Clinical Evidence

**DOI:** 10.3390/jcm14113983

**Published:** 2025-06-05

**Authors:** Wojciech Michał Glinkowski, Grzegorz Gut, Dariusz Śladowski

**Affiliations:** 1 Center of Excellence “TeleOrto” for Telediagnostics and Treatment of Disorders and Injuries of the Locomotor System, Department of Medical Informatics and Telemedicine, Medical University of Warsaw, 02-091 Warsaw, Poland; 2 Stichting MED PARTNERS, 1052 HK Amsterdam, The Netherlands; 3 Department of Transplantology and Central Tissue Bank, Medical University of Warsaw, 02-091 Warsaw, Poland; grzegorz.gut@wum.edu.pl

**Keywords:** platelet-rich plasma (PRP), knee osteoarthritis (KOA), regenerative medicine, intra-articular injection, hyaluronic acid, clinical efficacy, growth factors, leukocyte-poor PRP, pain management, orthobiologics

## Abstract

Background: Platelet-rich plasma (PRP) is increasingly utilized for managing knee osteoarthritis (KOA), yet its clinical value remains debated due to the variability in preparation protocols and outcome measures. Methods: This narrative review synthesizes current evidence from 40 high-quality studies published between 2013 and March 2025, including randomized controlled trials, systematic reviews, and meta-analyses. The biological mechanisms, clinical effectiveness, safety, and implementation challenges of PRP therapy in KOA are examined. Results: PRP injections—particularly leukocyte-poor PRP—demonstrate superior pain relief and functional improvement compared to hyaluronic acid and corticosteroids, especially in patients with mild to moderate KOA (Kellgren–Lawrence grades I–III). However, heterogeneity in PRP formulations (platelet/leukocyte content and activation protocols), injection regimens, and follow-up durations limits direct comparability across studies. Evidence from high-quality placebo-controlled trials shows inconsistent long-term benefits, with some failing to demonstrate superiority over saline beyond 6–12 months. The GRADE assessment rates the overall certainty of evidence as moderate. PRP appears safe, with few adverse events reported, but remains costly and variably reimbursed. Guidelines from major societies remain cautious or inconclusive. Conclusions: PRP is a promising, safe, and well-tolerated option for early to moderate KOA. However, the standardization of preparation protocols, patient selection criteria, and outcome reporting is essential to improve comparability and guide clinical practice.

## 1. Introduction

Knee osteoarthritis (KOA) is a highly prevalent and disabling joint disorder that leads to progressive articular cartilage degeneration, subchondral bone remodeling, and low-grade inflammation [1,2,3]. It poses a substantial burden on public health systems globally, and its incidence is projected to increase owing to aging populations and obesity [4]. Osteoarthritis (OA) is characterized by imbalanced catabolic and anabolic activities within joints, driven by oxidative stress, cellular senescence, and mechanical overload [5,6,7,8]. In addition to cartilage loss, OA [9] involves synovial inflammation, meniscal degeneration, bone marrow lesions, and osteophyte formation, all contributing to pain and functional limitation [2,5,10]. Platelet-rich plasma (PRP), an autologous product derived from centrifuged whole blood, has gained widespread interest as a regenerative treatment for musculoskeletal disorders [11,12]. PRP contains a concentrated mixture of platelets and bioactive growth factors capable of modulating inflammation, promoting tissue repair, and stimulating anabolic processes in chondrocytes, synoviocytes, and mesenchymal stem cells [13,14,15]. When injected intra-articularly, PRP may act on multiple OA-related targets, improving pain and potentially delaying disease progression [16,17]. Despite the increasing application of PRP in clinical settings, there is considerable variability in outcomes across studies [18,19]. This inconsistency stems from the heterogeneity of PRP formulations, preparation methods, platelet/leukocyte concentrations, activation protocols, and injection regimens [12,20,21]. Additionally, classification systems, such as platelet count, activation, white blood cells (PAW), dose, efficiency, purity, and activation (DEPA), have been proposed to improve PRP standardization and comparability. However, universal adoption remains limited [22,23,24].

This review summarizes the current understanding of platelet-rich plasma (PRP) applications in KOA by integrating mechanistic evidence, clinical outcomes, and recommendations. This review situates the reader within the broader context of OA treatment before identifying the key challenges in PRP-based interventions. The structured analysis of evidence from clinical trials, systematic reviews, and meta-analyses has enabled the evaluation of achievements in this field. This review critically evaluates the strengths and weaknesses of available data and identifies patterns across studies. Research gaps are discussed to delineate future investigation directions, including hypotheses worth pursuing. Special emphasis is placed on the safety profile of PRP, its relative efficacy compared to other intra-articular treatments (e.g., hyaluronic acid, corticosteroids, and placebo), and gaps in standardization and methodological rigor.

## 2. Materials and Methods

### 2.1. Study Design

A systematic search strategy enhanced the transparency and rigor of this study. The conclusions were drawn from clinical trials, foundational scientific papers, and expert consensus studies published between January 2013 and April 2025. A structured literature search was conducted across five major databases from January 2013 to March 2025. Initially, a direct and straightforward search yielded 134,464 records: PubMed (14,086), Web of Science Core Collection (2546), ScienceDirect (71,890), Cochrane Library (36 systematic reviews, six protocols, 9937 studies, and three clinical responses), and Embase (35,966). The search terms included (“platelet-rich plasma” OR PRP) AND (“osteoarthritis of the knee” OR “knee OA”) AND (“randomized controlled trial” OR “RCT” OR “meta-analysis” OR “systematic review” OR “mechanism”). Narrowing down the search phrases by applying filters to publications in English involving adult populations and clinical trials (RCTs, systematic reviews, and meta-analyses) significantly reduced the scope of the search results. Studies were deemed eligible if they met the following criteria: Studies were deemed eligible if they met the following criteria: population, adults with symptomatic KOA, intervention, intra-articular PRP injection (monotherapy), comparisons, placebo, hyaluronic acid (HA), corticosteroids, or other injectable agents, outcomes, pain relief, functional improvement, structural/biological changes, and safety profile. The study types included RCTs, systematic reviews, meta-analyses, and mechanistic studies. The exclusion criteria were publications in languages other than English, animal studies, in vitro studies, and studies lacking a specific PRP protocol or comparison group. Studies with follow-up periods of less than 6 months were excluded. All the publications with incomplete data have been excluded from the initial selection. The titles and abstracts were manually selected. The full texts were retrieved and assessed for methodological quality and relevance. Forty high-priority studies were selected based on methodological rigor, clinical significance, and direct relevance. Data extraction focused on study design, sample size, PRP preparation, injection protocol, follow-up duration, outcomes, pain/function measures, adverse events, and subgroup and sensitivity analyses.

### 2.2. Data Extraction

Participant Characteristics: The extracted data included the total sample size, the number of participants in each intervention group, age range or mean age, sex distribution, inclusion criteria (notably the Kellgren–Lawrence (KL) grade), and exclusion criteria. When detailed information is absent or ambiguous, the term ‘not reported’ should be employed for the relevant characteristics. Precise numerical values should be obtained whenever possible. Intervention details: For the PRP and HA groups, the number of injections, the frequency/schedule of injections, the volume of injections, specific preparation methods (if described), and additional treatment protocols were included. In cases where detailed information is unavailable, the term ‘not specified’ should be used. It is essential to provide as much detail as possible, including exact numbers and time intervals. Outcome Measures: All primary and secondary outcome measures utilized in the study should be listed, including specific assessment tools (e.g., the International Knee Documentation Committee Subjective Knee Form (IKDC), the Western Ontario and McMaster Universities Arthritis Index (WOMAC), and EuroQol Visual Analogue Scale (EQ VAS), measurement points, and specific scores or indices used for assessment. The primary outcome measures must be reported, as described in this study. If multiple outcome measures are employed, they should be listed in the order of priority, as indicated by the authors. Duration of Follow-up: Summary: The total duration of follow-up at specific time points (e.g., 2, 6, 12, and 24 months), the time point of the final assessment, and exact periods in units should be summarized. If multiple observation periods were specified, the patients were listed chronologically.

### 2.3. Synthesis

**Figure 1:**
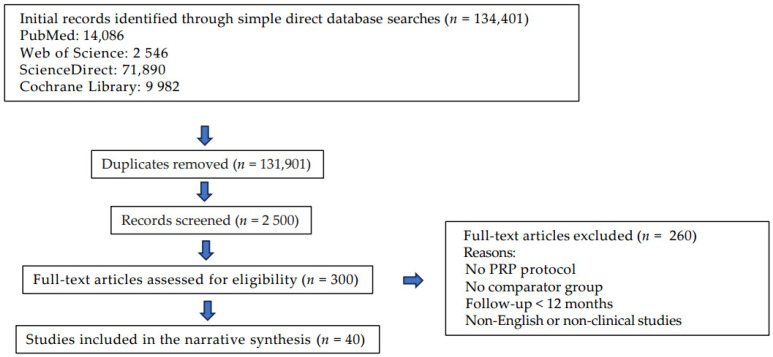
PRISMA flow diagram—Literature search and selection strategy. Final inclusion comprised 40 studies: 24 randomized controlled trials, 10 systematic reviews, and 6 meta-analyses.

Given the clinical heterogeneity of PRP preparations, such as Leukocyte-Poor PRP (LP-PRP) vs Leukocyte-Rich PRP (LR-PRP),and variations in the injection protocols and outcome measures, a narrative synthesis approach was used. The results were thematically categorized based on comparisons, including PRP vs. HA, corticosteroids, placebo, PRP composition, safety outcomes, and clinical guideline recommendations. Methodological tools to enhance transparency and rigor included a PRISMA-style flow diagram that illustrated the study selection process. The risk of bias (RoB) was assessed using the Cochrane criteria and summarized in tabular form. The certainty of the evidence was evaluated using the GRADE framework, with the findings presented in tabular and visual formats. An evidence map was developed to visualize the landscape of PRP preparations and comparators, highlighting areas of evidence concentration and identifying research gaps. The PRISMA flow diagram illustrates the study selection process (Figure 1).

## 3. Results

### 3.1. Summary of Included Studies

In total, 300 articles were identified as most pertinent to the query. Ultimately, 40 studies were selected for analysis, comprising 11 randomized controlled trials (RCTs) and 7 double-blind studies (Table 1). Twelve studies were systematic reviews, meta-analyses, or both. The sample size was reported in 32 of the 40 studies, with 25,144 participants. The sample size was not available in eight studies. The treatment protocol was reported in 20 of the 40 studies, with the most commonly reported protocol being three weekly injections, as noted in 10 studies. Other protocols included one, two, and four injections per week. The treatment protocol has not been described in the remaining 20 studies. The duration of follow-up, defined as the period during which information was available, was reported in 31 of the 40 studies, ranging from 3 months to 64.3 months. The most common follow-up duration reported in the 15 studies was 12 months. However, the duration of follow-up was not mentioned in nine studies.

### 3.2. Treatment Effects

The summary of PRP treatment effects after 3, 6, and 12 months is presented in Table 2.

The studies included in the analysis compared platelet-rich plasma (PRP) and hyaluronic acid (HA) treatment. PRP and HA showed significant improvements from the baseline at 3 and 6 months. However, at 12 months, PRP maintained its improvement, whereas HA showed less sustained improvement. Regarding between-group differences, PRP consistently outperformed HA at all three time points (3, 6, and 12 months). The weighted mean difference (WMD) or mean difference (MD) favored PRP: At 3 months, WMD = −0.25 (95% CI: −0.40 to −0.10, *p* = 0.0009); At 6 months, MD = −14.18 (95% CI: −26.12 to −2.23, *p* = 0.02); At 12 months, WMD = −0.64 (95% CI: −0.79 to −0.49, *p* < 0.00001). Upon analyzing the data collected over a 12-month period, it is challenging to assert that PRP demonstrates a definitive advantage. Furthermore, the data did not indicate any instances where HA was superior to PRP. Numerous studies failed to show a significant difference when compared to HA. A summary of the effects of PRP treatment compared to HA after 3, 6, and 12 months is presented in Table 3.

### 3.3. Functional Outcomes

The studies included in this analysis assessed functional outcomes at 3, 6, and 12 months post-treatment. For the PRP treatment group, a significant improvement from baseline was observed at both 3 and 6 months, with this improvement being sustained at 12 months. In contrast, the HA treatment group demonstrated improvement from baseline at 3 and 6 months; however, this improvement was less sustained at 12 months compared to the PRP group. When comparing the two groups, PRP consistently outperformed HA at all time points. At 3 and 6 months, the superiority of PRP was determined based on IKDC scores, whereas at 12 months, it was based on WOMAC functional scores. Specifically, PRP showed better outcomes (IKDC scores: weighted mean difference = 7.45; 95% CI, 2.50 to 12.40, *p* = 0.003) after 3 months, (IKDC scores: weighted mean difference = 5.06; 95% CI, 1.94 to 8.18, *p* = 0.001) after 6 months, and (WOMAC function: weighted mean difference = −7.69; 95% CI, −12.86 to −2.52, *p* = 0.004) after 12 months. All differences between the groups were statistically significant (*p* < 0.01). A summary of the functional results comparing PRP and HA after 3, 6, and 12 months is presented in Table 4.

### 3.4. Data on Adverse Events

Adverse events (AEs) associated with PRP treatment for KOA are predominantly mild, transient, and localized, with infrequent serious complications. The most common mild and transient AEs include post-injection pain flare-ups. Less frequent adverse events included swelling at the injection site and joint stiffness or tightness within 24–48 h. These effects are believed to result from PRP’s acute inflammatory response of PRP, particularly in leukocyte-rich PRP (LR-PRP) formulations. Typically, these AEs resolve within a few days and are managed with rest, cooling, and acetaminophen, as nonsteroidal anti-inflammatory drugs (NSAIDs) are often advised against post-PRP [62]. Infections such as septic arthritis are rare, and no cases have been reported in large trials such as RESTORE [63]. No cartilage damage, accelerated degeneration, evidence of tumorigenic effects, or abnormal calcification was observed. No significant systemic laboratory changes or systemic effects such as inflammation or clotting were observed because platelet activation was localized. Kim et al. [64] reported that leukocyte-poor PRP (LP-PRP) injections resulted in significantly fewer adverse events than LR-PRP. In most PRP studies, “adverse events” refer not to harmful reactions but to expected post-injection effects (e.g., mild flares), which are short-lived and manageable. PRP’s autologous nature and lack of synthetic additives for PRP significantly contribute to its favorable safety profile. Studies [28,33,48] evaluated the safety of PRP and HA injections and found mild and transient adverse events with both treatments. Khoshbin et al. [28] reported higher adverse events in the PRP vs. HA/placebo groups (8.4% vs. 3.8%, *p* = 0.002), mostly minor and short-lived events. Filardo et al. [33] reported that PRP caused more post-injection swelling and pain than HA. Tan et al. [48] found no significant difference in adverse events between the groups (relative risk: 1.21; 95% CI: 0.95–1.54, *p* = 0.13). Although PRP showed slightly more minor adverse events in some studies, both treatments had acceptable safety profiles.

Single vs. Multiple Injections: McLarnon and Heron [52] reported that triple PRP injections were superior to single injections over 12 months (*p* < 0.01). Tang et al. [47] found that PRP achieved better WOMAC scores with two injections. The optimal number and frequency of injections remain unclear and may depend on disease severity and patient characteristics.

### 3.5. Disease Severity

However, the effects of osteoarthritis severity have not been reported consistently. Cole et al. [65] found that patients with mild osteoarthritis showed significant improvement with PRP. Kon et al. [66] reported that PRP is more effective in younger patients with less cartilage degeneration. Other studies have found no significant differences based on disease severity. The relationship between the severity and outcomes remains unclear in these studies.

### 3.6. Risk of Bias Assessment

A thorough risk of bias (RoB) assessment was conducted on the main randomized controlled trials included in the review to evaluate methodological quality [67]. The Cochrane RoB 2.0 tool was used to assess five key domains: (1) bias from the randomization process, (2) deviations from intended interventions, (3) missing outcome data, (4) outcome measurements, and (5) the selection of the reported results [68]. Each domain was categorized as “low risk”, “some concerns”, or “high risk”. The overall risk of bias for each study was determined by synthesizing the evaluations at the domain level. Studies showing a high risk in one or more domains were considered to have high overall bias. The findings are summarized in a structured RoB table (Table 5), highlighting recurring methodological issues such as inadequate blinding, unclear allocation concealment, and incomplete reporting of primary outcomes. It has to be acknowledged that detailed PRP protocols were explicitly provided in only half of the studies (20 out of 40), and 9 studies failed to report follow-up durations, considerably reducing reproducibility and increasing the risk of bias.

### 3.7. GRADE Assessment

The Grading of Recommendations Assessment, Development, and Evaluation (GRADE) approach was used to evaluate the certainty of evidence regarding the primary outcomes of platelet-rich plasma (PRP) treatment for KOA. This framework examines five critical domains that might reduce confidence in the evidence: the risk of bias, inconsistency, indirectness, imprecision, and publication bias. Each outcome, including pain reduction, functional improvement, adverse events, and structural progression, was rated with high, moderate, low, or very low certainty. Initially, randomized controlled trials were considered to have high certainty, but ratings could be downgraded based on limitations in the five domains. The GRADE process was conducted independently for each outcome and is presented in a summary table and visual matrix format to aid interpretability for clinical decision making and guideline development (Table 6).

### 3.8. Evidence Map of PRP Formulations and Comparators

**Figure 2:**
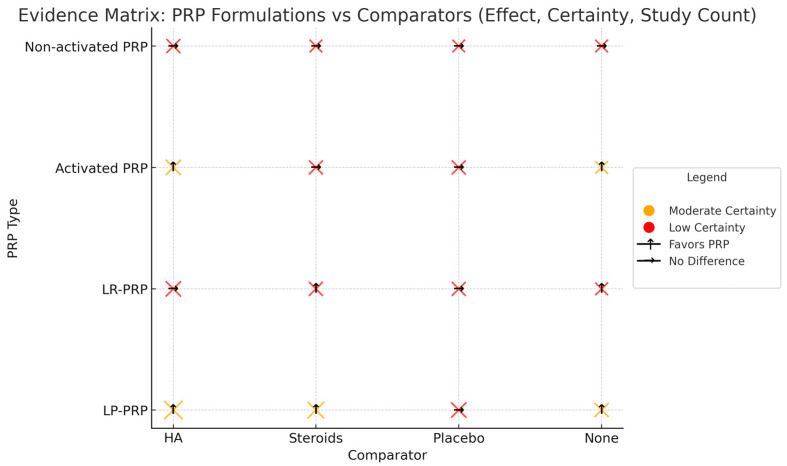
Evidence matrix—comparative analysis of PRP formulations vs. HA, steroids, and placebo. This matrix visually summarizes the clinical effect, GRADE certainty, and study volume for four types of platelet-rich plasma (PRP): leukocyte-poor PRP (LP-PRP), leukocyte-rich PRP (LR-PRP), activated PRP, and non-activated PRP. Comparators include hyaluronic acid (HA), corticosteroids, placebo, and single-arm trials (no comparator). The bubble size corresponds to the number of studies. The arrow direction represents the direction of clinical benefit (↑ favors PRP, → no difference). The color indicates GRADE certainty: orange = moderate, and red = low.

An integrated matrix comparing PRP preparations in terms of key clinical outcomes and comparative products has been developed to synthesize the current state of knowledge. Figure 2 shows the effects of individual preparations, their mode of action, and the strength of evidence supporting their efficacy. This also highlights that LP-PRP shows the most consistent and favorable results, particularly when compared to HA and steroids. In contrast, non-activated PRP and LR-PRP show more variable or uncertain results.

## 4. Discussion

This narrative review explores evidence potentially supporting platelet-rich plasma (PRP) as a promising therapeutic intervention for KOA undertaken in this narrative review. An analysis of randomized controlled trials and meta-analyses indicated that PRP was more effective than hyaluronic acid (HA) and corticosteroids in reducing pain and improving function over medium- and long-term follow-up. PRP benefits early to moderate OA (Kellgren–Lawrence grades I–III) and enhances the WOMAC and IKDC scores at 6 and 12 months. Studies have shown that repeated PRP injections following a three-weekly protocol offer more lasting benefits than single injections. These findings support the use of PRP as a treatment for KOA, particularly in mild-to-moderate cases. Clinical trials have shown improved patient-reported outcomes compared to hyaluronic acid or the placebo while maintaining a favorable safety profile. In vitro and preclinical studies have supported PRP’s biological benefits of PRP and demonstrated its anti-inflammatory, immunomodulatory, and regenerative potential. PRP delivers growth factors that modulate cartilage metabolism, influence synovial inflammation, and stimulate mesenchymal cell activity. These mechanisms align with OA’s multifactorial nature of OA and need for multimodal treatment. PRP’s regenerative potential of PRP stems from its growth factors, anti-inflammatory cytokines, and its ability to modulate the intra-articular environment. Clinical outcomes vary with the PRP preparation method, with leukocyte-poor PRP showing better tolerance than leukocyte-rich formulations. Despite positive trends, heterogeneity in PRP formulations, injection protocols, and outcome measurements limits the generalizability of the results. Some high-quality trials have shown no significant advantage over the placebo, indicating the need for personalized treatments. Safety outcomes remain favorable, with most adverse events being mild and self-limiting, supporting PRP as a safe alternative to corticosteroids and HA in younger active patients.

### 4.1. Mechanisms of Action of PRP in Osteoarthritis

The therapeutic rationale for PRP in osteoarthritis relies on the high concentration of growth factors and bioactive molecules in platelets. When injected into the knee joint, platelets are activated by collagen exposure or external activators, such as calcium or thrombin, which release growth factors from alpha granules. Key factors include transforming growth factor-beta (TGF-β), platelet-derived growth factor (PDGF), insulin-like growth factor-1 (IGF-1), vascular endothelial growth factor (VEGF), and basic fibroblast growth factor (bFGF). These factors stimulate chondrocyte anabolic activity and synoviocyte function, enhancing cartilage matrix synthesis and joint lubrication [70]. PRP contains cytokines and chemokines that modulate the inflammatory environment of the osteoarthritic joint [71]. PRP increases hyaluronic acid synthesis by synovial cells and suppresses catabolic mediators like matrix metalloproteinases (MMPs), which contribute to cartilage breakdown [71]. These effects provide the mechanisms for symptom relief and cartilage protection.

Zhuo et al. [72] showed that PRP counteracts the effects of inflammatory cytokines on chondrocytes. In human articular chondrocytes, PRP treatment reversed interleukin-1β (IL-1β)-induced inflammation, apoptosis, and extracellular matrix degradation [72]. PRP downregulates T-box transcription factor 3 (TBX3), a mediator of IL-1β catabolic effects on the cartilage. By inhibiting TBX3, PRP reduces NLRP3/caspase-1 inflammasome pathway activation and MMP-13 levels, thereby protecting chondrocytes from IL-1β damage [72]. These findings indicated that PRP induces an anti-inflammatory pro-survival shift in the joint microenvironment. Studies have also shown that PRP injections decrease synovial membrane inflammation and slow cartilage degeneration compared with controls [73]. Growth factors in PRP may recruit reparative cells, such as resident mesenchymal stem cells, to injury sites and enhance their proliferation into cartilage-forming cells. However, this hypothesis requires further investigation.

**Figure 3:**
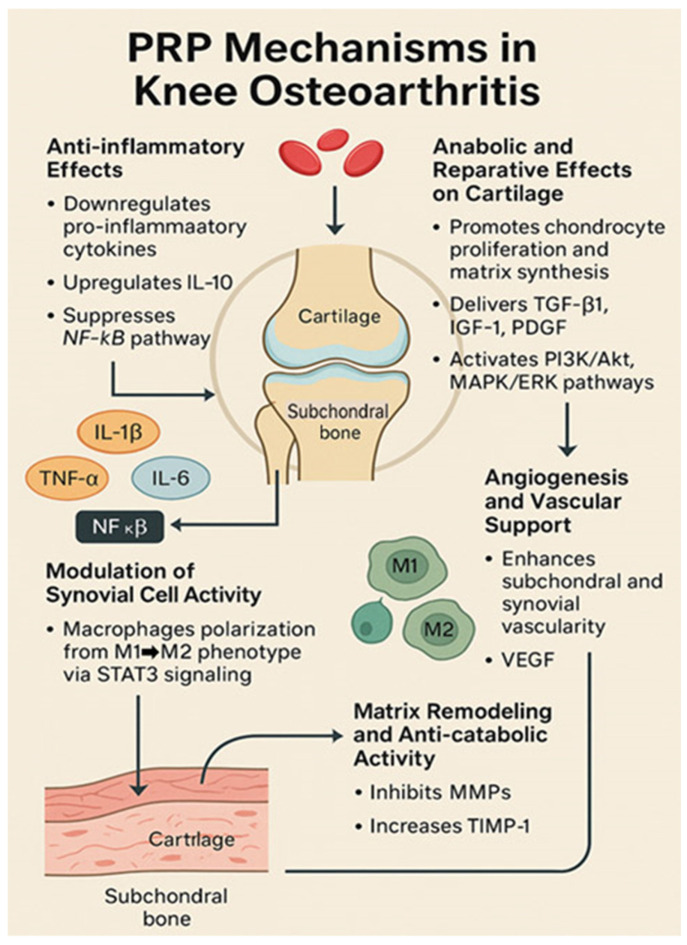
Mechanisms of action of PRP in osteoarthritis.

PRP’s effects of PRP on PRP vary depending on its composition and joint conditions. Osteoarthritic joints show an imbalance between pro-inflammatory and anti-inflammatory factors, and PRP helps to restore equilibrium. PRP inhibits pro-inflammatory mediators such as IL-1β and tumor necrosis factor-alpha (TNF-α) while enhancing anti-inflammatory signals [71]. PRP may alleviate pain through structural modifications and neuromodulation by reducing the levels of inflammatory mediators that sensitize the pain receptors. Studies have shown that PRP can lower neuropeptides such as substance P in joint tissues, correlating with pain relief [74]. While the mechanism remains complex, PRP’s bioactive profile of PRP suggests actions that promote tissue regeneration, inhibit inflammation, and slow osteoarthritis progression [71]. These findings support the clinical use of PRP in patients with KOA. The mechanisms of PRP action in osteoarthritis are summarized in Figure 3.

### 4.2. PRP Preparation and Formulations

A critical aspect of platelet-rich plasma (PRP) therapy is the variability in preparation methods, which yield different concentrations of platelets, leukocytes (white blood cells), and plasma components. This lack of standardization affects the research and clinical outcomes. PRP is obtained by drawing a patient’s blood (often 15–60 mL) and centrifuging it to separate its components by density. The buffy coat layer and the plasma supernatant (containing platelets) were collected. This concentrate has platelet counts 3–5 times higher than baseline blood levels, although some systems achieve higher concentrations. A key variable is whether PRP preparation includes leukocytes (leukocyte-rich PRP, LR-PRP) or excludes them (leukocyte-poor PRP, LP-PRP). Leukocytes, particularly neutrophils, carry pro-inflammatory enzymes and reactive oxygen species that can cause joint inflammation. However, leukocytes may aid in tissue healing and low-grade joint infections. The debate over LR-PRP and LP-PRP remains prominent. In vitro studies suggest that high leukocyte content in LR-PRP may increase inflammation in the joint. High leukocyte PRP increases the levels of inflammatory cytokines, such as IL-1 and TNF-α, and catabolic enzymes in cell culture models [75]. A systematic review by Kim et al. (2021) found that leukocyte-poor PRP showed better outcomes in KOA than leukocyte-rich PRP; LP-PRP provided significant pain and functional improvements compared to controls (saline or hyaluronic acid). LR-PRP did not show a substantial benefit in some measures [76]. The review noted fewer adverse reactions with LP-PRP, suggesting that high concentrations of LR-PRP neutrophils might cause more post-injection pain or swelling [76]. These findings have led clinicians to favor leukocyte-poor formulations for intra-articular use.

There is no consensus on whether leukocyte-poor platelet-rich plasma (LP-PRP) is superior [77,78]. Some studies have suggested that leukocytes in PRP might promote regeneration through macrophages, adopting an anti-inflammatory role [79,80]. Researchers argue that leukocyte-rich PRP (LR-PRP) can enhance healing by leveraging neutrophil-platelet interactions that produce lipid mediators, aiding inflammation resolution [81]. A double-blind randomized controlled trial (RCT) by Di Martino et al. [82] compared LR-PRP and LP-PRP in patients with KOA patients. In this study, 90 patients received LR-PRP and 85 received LP-PRP, with three injections at one-week intervals, followed by a 12-month observation period [82]. The results showed no significant difference in clinical outcomes; both groups had similar pain and function score improvements, with comparable patient satisfaction at one year [82]. However, the LR-PRP group showed a higher incidence of transient post-injection pain flares and swelling, suggesting that leukocytes provoke a stronger inflammatory response [82]. No serious adverse events occurred in either of the groups. Although the efficacy was equivalent, LP-PRP showed better tolerability. Another consideration is whether platelets are exogenously activated before injection for immediate growth factor release or in the native state. A meta-analysis suggested that exogenously activated PRP might improve pain and function more than non-activated PRP in patients with knee OA, although further research is needed [83]. The number and timing of injections varied between single and multiple administrations spaced one–two weeks apart, with no consensus on the optimal regimen. Most RCTs used two- or three-injection series. A network meta-analysis by Qiao et al. found that approximately 10 trials used a single injection, and up to three other trials had improved outcomes over one–three months regardless of the number of injections, although the benefits of multiple injections remain unclear [84]. Variations in the PRP formulation and administration schedule may explain the heterogeneous study results. Clinicians should use standardized high-quality PRP preparations and follow validated protocols to achieve optimal outcomes.

### 4.3. Clinical Efficacy of PRP in KOA

Randomized trials of knee OA have evaluated intra-articular platelet-rich plasma (PRP) injections in patients with KOA. These trials compared PRP with controls, including the placebo (saline), corticosteroids, hyaluronic acid (HA), and other biological treatments. The patient population consisted of individuals with mild-to-moderate OA (Kellgren–Lawrence grades I–III) who retained the joint space. Below, we summarize the key findings from these trials by the type of comparison.

#### 4.3.1. PRP vs. Placebo (Saline)

High-quality randomized controlled trials (RCTs), such as the RESTORE trial [63] and Dório et al. [62], reported no significant difference between PRP and the placebo in alleviating knee OA pain or preventing cartilage loss at 12 months. These findings suggest that the benefits of PRP may not surpass those of the placebo in specific populations. Placebo-controlled trials are essential for determining injection efficacy beyond the placebo effect. However, small placebo-controlled RCTs have produced mixed results. A pilot study by Patel et al. [69], which predated our 10-year focus, suggested that PRP might be superior to saline, although this was not definitive [69]. Recently, large high-quality trials have been conducted. The RESTORE trial by Bennell et al. [63] provides the most robust evidence. In this multicenter trial, 288 patients with mild-to-moderate knee OA received three weekly PRP or placebo (saline) injections, with outcomes assessed over 12 months [63]. The results showed no statistically significant differences between PRP and the placebo in terms of primary outcomes. After one year, knee pain (measured by a 0–10 numeric rating scale) improved by −2.1 points in the PRP group compared to −1.8 in the saline group, a non-significant difference [63]. The function measures showed similar improvements in both groups. The MRI-based assessment of medial tibial cartilage volume revealed no significant difference in cartilage loss between PRP and saline; both groups experienced slight cartilage loss over 12 months, indicating that PRP did not halt structural progression relative to the placebo [63]. This rigorous trial indicates that the benefits attributed to PRP in previous studies may have been influenced by the placebo effect or other non-specific effects associated with injections. The authors observed that clinical guidelines at the time did not endorse PRP because of insufficient conclusive evidence, and their findings supported a cautious approach [63]. Similarly, a smaller placebo-controlled RCT conducted by Dório et al. [62] compared PRP to saline. Two injections were administered two weeks apart in this double-blind trial involving 62 participants (PRP, *n* = 20; saline, *n* = 21; and exploratory third arm of plain plasma, *n* = 21) [62]. At the 24-week follow-up, no significant differences in pain or functional outcomes were observed between the PRP and saline placebo groups. All groups, including the placebo group, reported modest pain relief (~30–35% improvement in VAS pain from baseline), but PRP did not demonstrate superiority over saline [62]. Secondary measures, such as the Knee Injury and Osteoarthritis Outcome Score (KOOS) and the timed get-up-and-go test, also showed no differences among the groups. Notably, PRP-treated patients experienced more post-injection localized adverse events (increased pain or swelling) than the saline group (65% vs. 33% of patients, respectively), although these events were transient [62]. This trial corroborated the RESTORE findings by concluding that PRP did not offer a clear advantage over placebo injections in the short- to mid-term. Importantly, none of the placebo trials yielded any negative results. Some studies have indicated trends favoring PRP, particularly at specific time points, but the general consensus from high-quality designs is that PRP’s benefits of PRP over saline are modest. Placebo-controlled trials [49,85] have found that, while PRP may provide slight improvements in some scores at intermediate follow-ups (e.g., 3 or 6 months), these differences often become statistically insignificant by 12 months or are of questionable clinical significance [83]. For instance, one study reported that PRP improved WOMAC pain by a few points compared with saline at 3–6 months; however, by 12 months, this advantage diminished [83]. These findings suggest that if PRP is effective, its effects may manifest in the earlier post-injection period, and placebo effects may be significant in any injection-based intervention for knee OA.

#### 4.3.2. PRP vs. Hyaluronic Acid

Some RCTs and meta-analyses [37,49] have reported superior outcomes with PRP compared to HA, particularly at longer follow-up periods (6–12 months). PRP showed greater improvements in WOMAC pain and function scores with more sustained effects than HA. Hyaluronic acid (HA) injections are another standard treatment for knee OA. Multiple RCTs have compared PRP with HA because both aim to relieve symptoms and improve joint function. These trials have favored PRP, especially over longer periods. A landmark trial by Patel et al. (2013) found that PRP resulted in better pain and function scores at six months than HA [69]. Dai et al. (2017) conducted a meta-analysis of 10 RCTs (1069 patients) that compared PRP with control injections (mostly HA). They found that PRP and HA provided similar pain relief at six months post-injection. However, at 12 months, PRP showed significantly greater improvements in pain and function than HA [37]. Pooled analysis at 12 months showed that PRP improved WOMAC pain scores more than HA (mean difference ~ −2.8 points, *p* = 0.0001) and WOMAC function scores (mean difference ~ −12.5 points, *p* < 0.00001), exceeding the minimum clinically significant difference [37]. After one year, patients who received PRP had less pain and a better functional status than those who received HA. The outcomes at 6 months were more equivalent, indicating that the effects of PRP might be longer-lasting. PRP’s 12-month benefit was also observed vs. saline, showing superior pain and function results at 6 and 12 months [37]. These results suggested that PRP may be more effective than viscosupplementation in the intermediate term.

Since 2015, numerous randomized controlled trials (RCTs) have confirmed the benefits of platelet-rich plasma (PRP) over hyaluronic acid (HA). For example, in a randomized trial, Shen et al. (2017) found that PRP led to a more significant pain reduction than HA at a 6-month follow-up [38]. Similarly, Murali et al. [86] noted that PRP provided longer-lasting symptom relief than a single HA injection [54]. A comprehensive network meta-analysis by Qiao et al. (2023) synthesized data from 35 RCTs involving over 3100 patients to compare PRP, HA, corticosteroids, saline, and combination treatments. This network analysis ranked PRP as the most effective intervention for KOA patients. At the 3-month mark, PRP, especially when combined with HA, was linked to superior Western Ontario and McMaster Universities Osteoarthritis Index (WOMAC) scores [84]. By 6 months, PRP alone showed significantly better pain relief than HA or corticosteroids [84]. For instance, a network meta-analysis indicated that PRP improved WOMAC pain scores more than HA, with a mean difference ranging from approximately −3 to −6 points at 6 months, depending on the analysis, which was statistically significant [84]. Although these differences are not exceedingly large, they may be clinically significant in patients. At 12 months, PRP continued to outperform HA in terms of pain and functional outcomes in most studies, although fewer trials extended to a 1-year duration. The consistent benefits of PRP across multiple independent trials and meta-analyses have led the authors to conclude that PRP injections yield equal or greater clinical improvement than HA injections in KOA [37,84]. Furthermore, the safety profiles of PRP and HA were comparable, with no increase in serious adverse events observed in patients treated with PRP [37].

#### 4.3.3. PRP vs. Corticosteroids

Meta-analyses, such as Tiwari et al. [87], showed that platelet-rich plasma (PRP) provides longer symptom relief than corticosteroid injections, which lose effectiveness after 4–6 weeks. PRP outperformed corticosteroids in terms of pain and functional outcomes at 3 and 6 months post-injection. Intra-articular corticosteroid injections, including triamcinolone, effectively relieve short-term pain and are beneficial after 4–6 weeks. Frequent steroid injections are discouraged because of the potential cartilage damage and systemic effects. PRP has a slower onset but a longer duration of action. Trials comparing PRP with steroid injections indicate that PRP may not initially provide immediate pain relief like steroids; however, PRP yields superior outcomes by 2–3 months and beyond. Raeissadat et al. [31] found that at one month, steroids were more effective for pain relief than PRP; however, at three and six months, the PRP group experienced less pain than the steroid group [31]. The network meta-analysis by Qiao et al. [84] showed that at 6 months, PRP was more effective than steroids in improving WOMAC pain (mean difference ~–8.1 points, *p* = 0.004) and VAS pain (MD ~ −1.11 cm, *p* < 0.001) [84]. The most pronounced difference was observed 6 months post-injection, after which steroid benefits dissipated, whereas PRP benefits persisted. By 12 months, the steroid groups had typically returned to baseline symptoms, whereas the PRP groups maintained their improvement.

Similarly, a meta-analysis by Tiwari et al. in 2022 [87] concluded that platelet-rich plasma (PRP) provides more sustained pain relief than corticosteroid injections in managing KOA, especially after three months. While corticosteroids remain relevant for acute flare-ups, data suggest that PRP may be beneficial for long-term symptom control. Notably, frequent steroid injections have been linked to potential cartilage thinning over time [88]. However, PRP does not pose this concern and may even exert protective effects. However, it should be noted that PRP does not definitively show structural modifications in trials such as RESTORE [63]. In summary, evidence from randomized controlled trials (RCTs) indicates that PRP produces outcomes comparable to or better than those of steroid injections beyond the initial one–two months, supporting the notion that PRP addresses the underlying joint environmental factors rather than merely providing transient inflammation blockade.

#### 4.3.4. PRP vs. Other Biologics (e.g., BMAC)

Evidence from limited head-to-head trials suggests that platelet-rich plasma (PRP) is as effective as bone marrow aspirate concentrate (BMAC) while being easier to prepare and more cost-effective, making it a practical first-line biological injectable. Several studies have compared PRP with other emerging injectables such as BMAC, which contains stem cells and growth factors. To date, these comparisons have generally not identified a definitive superior treatment; PRP and BMAC seem to have similar efficacy in managing KOA. For example, one randomized trial [89] reported no significant difference in outcomes between BMAC and PRP at 12 months, with both groups showing modest improvement and no superiority in cell-based treatments. A systematic review by Elksnis and Vallence [90] observed that both PRP and BMAC improved pain and function relative to the baseline, although few head-to-head trials are available. Given the easier preparation and lower cost of PRP compared to BMAC, some clinicians prefer PRP as the initial treatment. Indeed, a scoping review highlighted that PRP results in knee OA that is “clinically comparable or superior” to other injectable options, including BMAC [91,92]. Thus, the current evidence suggests that PRP is a viable alternative to other orthobiologics.

### 4.4. Magnitude of Clinical Effects of PRP

To effectively evaluate the typical improvements offered by platelet-rich plasma (PRP) therapy, pain reduction metrics should be considered. Numerous clinical trials have employed a 0–10 pain scale or the Western Ontario and McMaster Universities Osteoarthritis Index (WOMAC) on a 0–100 scale. Patients receiving PRP treatment often report pain reductions of 2–3 points on the 0–10 scale and 10–20 points on the WOMAC scale over 6–12 months [37,63]. These reductions met or exceeded the minimal clinically significant differences observed in several patients. For example, a meta-analysis by Filardo et al. [33] found that approximately 60% of the patients experienced clinically relevant improvement at the 6-month follow-up. In contrast, placebo injections have been shown to alleviate these symptoms. Bennell et al. [14] demonstrated that the saline group showed an improvement of approximately 1.8 points out of 10, underscoring the placebo effect and natural symptom fluctuations associated with osteoarthritis [63]. Consequently, while PRP does not “cure” osteoarthritis or regenerate normal cartilage, it can significantly alleviate symptoms for many patients, particularly in the short- to medium-term. The challenge, as evidenced, is that by one year, the disease may progress, and symptoms may reemerge, suggesting that PRP might need to be repeated or combined with other therapies for sustained disease management.

### 4.5. Systematic Reviews and Meta-Analyses

Over the past decade, numerous systematic reviews and meta-analyses have been conducted to synthesize an expanding body of evidence. These analyses vary in both scope and quality; some focus on platelet-rich plasma (PRP) compared to a specific comparator such as hyaluronic acid (HA), whereas others include all randomized controlled trials (RCTs) against any control, and some use network meta-analytic techniques to indirectly compare multiple treatments. Collectively, these reviews have generally reached favorable conclusions regarding the efficacy of PRP, although they caution about study heterogeneity and the risk of bias in certain trials. A comprehensive review of meta-analyses by Mende et al. [93] evaluated 39 published systematic reviews, including seven network meta-analyses, on PRP for KOA. This higher-order summary revealed that the majority of reviews (17 publications) concluded that PRP offers clear benefits for knee OA, with an additional 3 suggesting possible efficacy and 8 recommending PRP as a treatment option [93]. Across these meta-analyses, PRP has consistently been reported to significantly alleviate pain and enhance the function, stiffness, and quality of life of patients with knee OA within 12 months of treatment [93]. Several reviews have reported that the adverse effects of PRP are minor and transient. Notably, in several network meta-analyses comparing multiple injection therapies, PRP was ranked favorably. This study highlighted that in the surface under the cumulative ranking (SUCRA) analysis, PRP frequently appeared among the top three treatments for knee OA outcomes, with both leukocyte-poor PRP (LP-PRP) and leukocyte-rich PRP (LR-PRP) ranking within the top four treatment options [93]. This evidence has gradually shifted the tone of the literature to support PRP more strongly in contrast to earlier skepticism.

A meta-analysis of randomized controlled trials (RCTs) demonstrated that platelet-rich plasma (PRP) significantly alleviated pain at 3, 6, and 12 months [85]. In contrast, saline/placebo treatments did not produce similar results, highlighting PRP’s superior effects over the placebo. A study by Xiong et al. [94], who analyzed 14 RCTs, revealed that PRP yielded better outcomes than hyaluronic acid (HA) in terms of pain, stiffness, and function at 6 and 12 months. Network meta-analyses that compared multiple treatments that were not directly tested within trials further supported these findings. A network meta-analysis by Qiao et al. [84] identified PRP, particularly leukocyte-poor PRP, as one of the most effective options for the treatment of knee OA. They found that PRP outperformed corticosteroids and HA in pain and function outcomes for up to one year, with PRP and HA combinations showing early advantages [84]. Similarly, a network meta-analysis by Khalid et al. [95] involving 42 RCTs and 3696 patients confirmed that PRP provided better pain relief than HA and corticosteroids at a 6-month follow-up [95]. PRP improved Western Ontario and McMaster Universities Osteoarthritis Index (WOMAC) pain scores more than HA (mean difference [MD] = −0.74, normalized 0–10 scale, *p* < 0.00001) and steroids (MD = −8.06, WOMAC pain [0–100 scale], *p* = 0.004), with sustained effects at 12 months [95].

While most reviews favor PRP, there is heterogeneity in the outcomes. Not all studies have shown benefits, and patient subgroups may respond differently. Factors such as OA severity, patient age, PRP formulation, and the injection technique may have influenced the results. Some studies have attempted to perform subgroup assessments. A meta-analysis suggested that younger patients and those with less severe OA responded better to PRP because early disease may have greater repair capacity [38]. However, the evidence remains inconclusive; some trials with patients with moderate OA showed benefits, while those with mild OA might benefit less from mild symptoms. Another systematic review found conflicting results on whether the OA grade correlates with PRP efficacy; some studies found PRP to be most effective in early OA, whereas others reported that late-stage patients experienced pain relief [96]. This underscores the need for research on the response predictors.

Meta-analyses consistently underscored the generally favorable safety profile of PRP. For example, Kim et al. [76], in their meta-analysis comparing LP and LR PRP, found that pooled adverse event rates, mainly mild flares, were low, with no studies reporting significant complications such as infection attributable to PRP. This review noted that LP-PRP significantly improved WOMAC scores compared to HA or the placebo, whereas LR-PRP did not, suggesting that leukocyte concentration may affect PRP’s efficacy [76]. However, a subsequent Di Martino RCT showed that both formulations were effective if prepared correctly, indicating the need for further data [82]. When synthesizing all available evidence, systematic reviews and meta-analyses from the past decade have generally asserted that PRP injections can lead to clinically significant improvements in pain, stiffness, and function in KOA, with effects lasting up to approximately 12 months [85,86,93,95,97,98]. PRP often surpasses or matches the benefits of other injectable therapies, such as hyaluronic acid and corticosteroids, during mid- to long-term follow-up. Nevertheless, the evidence is not without its limitations. There was variability among the studies, some risk of bias (as some early RCTs were not double-blinded), and potential publication bias (trials with positive results may have been published more readily than those with null results). The influence of the PRP preparation method complicates our conclusions. Therefore, while meta-analyses strongly endorse the use of PRP, they also advocate for standardization and further high-quality trials to optimize PRP treatment protocols and confirm long-term benefits.

### 4.6. Impact of PRP Formulation

#### 4.6.1. Leukocyte Content (LR-PRP vs. LP-PRP)

Di Martino et al. [82] and Kim et al. [76] compared leukocyte-rich (LR-PRP) and leukocyte-poor (LP-PRP) formulations. Although their efficacy was similar, LP-PRP demonstrated a better safety profile with fewer post-injection flares, leading many clinicians to prefer LP-PRP for intra-articular use.

#### 4.6.2. Platelet Activation

Meta-analyses [83] have suggested that exogenously activated PRP may yield superior clinical outcomes compared to non-activated formulations, although the data remain inconclusive.

### 4.7. Safety and Adverse Events

Platelet-rich plasma (PRP) has demonstrated good tolerability across several studies. The most common side effects were mild post-injection pain and swelling. No serious adverse events, such as infection or cartilage damage, were observed in large trials such as RESTORE. Leukocyte-poor PRP (LP-PRP) is associated with fewer adverse events than leukocyte-rich PRP (LR-PRP). The key benefit of PRP as an autologous treatment is its safe profile. Because PRP originates from the patient’s own blood, immunologic reactions or allergies are virtually nonexistent, unlike some avian-derived viscosupplements. PRP does not contain synthetic chemicals or exogenous drugs which result in minimal systemic effects. The most common adverse effects of PRP knee injections are localized and transient. Patients experienced mild pain flares, swelling, or stiffness in the injected knee within 24–48 h post-injection. This results from the acute inflammatory response triggered by PRP, particularly in the presence of leukocytes. Dório et al. [62] noted that 65% of PRP patients experienced transient knee pain following injection compared with 33% of saline placebo patients. These flares typically resolve within a week and are managed with rest, ice, and acetaminophen; nonsteroidal anti-inflammatory drugs (NSAIDs) are avoided post-PRP because inflammation is a part of healing. Kim et al. [76] found that LP-PRP injections resulted in fewer post-injection reactions than LR-PRP injections, supporting the hypothesis that reducing leukocytes improves tolerability. Serious adverse events associated with PRP use are rare. Although infection is a concern with any type of injection, sterile techniques are required. Cases of joint infections following PRP injections have only been documented in isolated instances across thousands of injections. No infections were noted in the RESTORE trial [14] or other significant trials. A meta-analysis showed no considerable difference in adverse events between the PRP and control groups [18]. Studies [63] have shown that PRP does not accelerate cartilage loss and have confirmed no difference in MRI cartilage outcomes between PRP and saline, and no joint deterioration due to PRP has been found [14]. This is in contrast with repeated steroid injections, which are linked to cartilage thinning over time [26].

One safety concern is the theoretical risk that platelet-rich plasma (PRP) could worsen inflammation in patients with severe osteoarthritis (OA) and inflamed joints. However, studies have not documented harmful effects in advanced cases; at worst, the patients may not improve. As PRP is an “orthobiologic”, some patients worry about “growth factors” potentially promoting abnormal tissue growth. Nevertheless, no evidence suggests that PRP induces tumorigenic effects or abnormal joint calcification. Growth factors can act locally and transiently. Another consideration is whether PRP causes systemic effects such as increased inflammation markers or coagulability. Research has shown that these effects are localized, with platelet activation at the injection site and released factors remaining within the joint tissues. Blood tests of PRP-treated patients showed no significant systemic changes in inflammatory markers [31]. PRP injections are generally more uncomfortable than steroid or hyaluronic acid (HA) injections because of their larger volumes (6–8 mL of PRP vs. 2–4 mL of HA) and potential flares. Patients should minimize strenuous activity in the injected knee for 1–2 days before resuming normal activity. Protocols recommend avoiding nonsteroidal anti-inflammatory drugs (NSAIDs) for one–two weeks after the injection to prevent blunting PRP’s action, although acetaminophen or mild opioids may be used for post-injection pain. No long-term restrictions are imposed. As PRP is autologous unlike pooled blood products, disease transmission is not a concern. Each patient’s PRP was used only on themselves to eliminate contamination risks. PRP is considered safe for knee OA, and its effects are limited to temporary local discomfort. This safety profile, without systemic side effects (such as hyperglycemia or blood pressure increase from steroid injections), makes PRP appealing, particularly for patients intolerant of other medications.

### 4.8. Practical Considerations and Implementation

In clinical practice, the use of platelet-rich plasma (PRP) in KOA involves several technical considerations. Patient Selection: Ideal candidates were individuals with mild-to-moderate osteoarthritis who lacked relief from conservative treatments and were either not ready for surgery or wished to postpone it. PRP is often offered to relatively young or active patients aged 40–65 years with a preserved joint space. Although older patients may consider PRP, those with advanced bone-on-bone arthritis may experience limited benefit. Physicians must ensure that there are no contraindications such as active infections or bleeding disorders. Patients receiving anticoagulation therapy may require adjustments based on blood draws and injections. Blood Draw and Preparation: Blood samples (30–60 mL) were drawn from the peripheral vein. Blood was processed using a PRP kit or a centrifuge system, with systems requiring distinct spin protocols. After centrifugation, the clinician isolated the platelet-rich fraction. The final PRP injection volume was 3–8 mL. Some systems yield highly concentrated PRP (up to 7–10 times the baseline platelet count) in small volumes, whereas others produce moderate concentrations in larger volumes. Consistency in preparation helps achieve predictable results. Injection Technique: PRP was administered intra-articularly using a sterile technique. Many practitioners use ultrasound guidance for accurate delivery, especially in knees with irregular anatomy or obese patients. Ultrasound-guided injections enhance precision, although landmark-based injections are sufficient for the knee joint space. PRP flowed well through a standard 22-gauge needle. It is essential to numb the skin and administer slow injections to minimize discomfort. Post-Injection Protocol: Unlike steroid injections, PRP does not provide immediate relief and may cause short-term soreness. Patients should rest for one or two days. Clinicians recommend avoiding anti-inflammatory medications (NSAIDs) for 1–2 weeks post-injection to allow the PRP-induced inflammatory cascade to proceed [44]. Physical therapy is often paused temporarily and resumed later. A recent review by Orchard et al. highlighted the absence of a standardized post-PRP rehabilitation protocol. Gentle range-of-motion and low-impact activities can be initiated within a week [99].

#### Repeat Treatments

The debate over PRP injection repetition continues. Many studies have focused on a single-injection series administered over three weeks. For patients who initially respond well but experience symptom recurrence after 9–12 months, clinicians may suggest a second round of PRP. There is no evidence of harm from repeated treatments; due to its safety, some patients opt for annual PRP injections for maintenance. However, repeated treatment costs were significant. Cost and Access: In many countries, insurance does not cover PRP therapy for osteoarthritis, as it is experimental or lacks proven long-term benefits. Patients face out-of-pocket expenses ranging from hundreds to thousands of dollars per injection, depending on the healthcare setting and PRP system. This cost may limit the access to PRP [100]. Cost-effectiveness analyses by Kon et al. [23] compared PRP with alternatives; if PRP delays knee replacement, it may prove cost-effective in the long-term, although more data are needed [66]. Regulatory Status: In the United States and many other regions, PRP is not FDA-approved as an osteoarthritis drug, but it is allowed as a procedure because it is classified as minimally manipulated autologous tissue. Clinics must administer PRP to the same patient from whom blood was drawn, typically on the same day, to comply with regulations. The PRP cannot be banked without appropriate licensing. Patient Counseling: Clinicians must set realistic expectations. Patients should be aware that PRP aims to alleviate symptoms and improve function, but it is not a cure or guarantee against future surgery. While some patients experience significant improvement, others may experience modest or no improvement. Given the variable outcomes, the individual responses differ. Patients often ask how PRP compares to alternatives such as hyaluronic acid (HA) or corticosteroid injections. Clinicians can explain that while PRP’s effects of PRP may take longer to manifest, they may be more enduring, addressing joint biology rather than providing temporary inflammation relief.

Interest in combining platelet-rich plasma (PRP) with other therapies is increasing. One method involves administering PRP and hyaluronic acid together (“PRP-HA”), with studies suggesting potential synergistic effects [48]. Another approach combines PRP injections with physical therapy or offloading braces to enhance the outcomes. PRP has also been used in arthroscopic debridement. Although these combination therapies have not shown clear superiority, they highlight ongoing innovation [63]. PRP for KOA should be a part of a comprehensive management strategy in clinical practice. Nonpharmacological interventions, such as exercise, weight management, and muscle strengthening, remain crucial alongside PRP therapy. PRP is not meant to replace these foundational treatments; it is meant to act as an adjunct that may improve outcomes. Successful PRP treatment may delay the need for invasive procedures such as osteotomy or total knee replacement, especially in younger patients. If PRP proves ineffective, patients can still pursue alternative treatments as PRP generally does not limit other therapeutic options.

### 4.9. Current Guidelines and Recommendations

Despite growing evidence of the symptomatic benefits of platelet-rich plasma (PRP) injections for knee osteoarthritis (KOA), major clinical guidelines advise caution in routine use (Table 7). This stems from the variability in study methodologies, a lack of standardized PRP preparations, and insufficient data on long-term effects. Since 2019, the American College of Rheumatology (ACR) and the Arthritis Foundation (AF) have recommended PRP injections for knee and hip osteoarthritis [101]. This recommendation is based on limited high-quality randomized controlled trial (RCT) evidence and concerns regarding cost and standardization. The Osteoarthritis Research Society International (OARSI) [102,103] Guidelines classified PRP as an “uncertain” treatment for knee osteoarthritis, noting positive symptomatic outcomes but citing high variability in trial designs. The American Academy of Orthopaedic Surgeons (AAOS) [104,105] guidelines concluded that evidence supporting PRP use remains inconclusive due to inconsistent results and a lack of standardized protocols. The VA/DoD 2020 Clinical Practice Guideline [106] advised against routine PRP use, stating that the evidence was insufficient for endorsement as a first-line treatment. European and international expert groups have adopted more favorable perspectives. The ESSKA ORBIT 2024 [107] consensus supports PRP for early to moderate osteoarthritis (Kellgren–Lawrence grades I–III). The GRIP 2020 [108] consensus endorsed PRP as an effective second-line therapy after conventional treatments fail, recommending one to three leukocyte-poor PRP injections under ultrasound guidance. The International Cartilage Regeneration and Joint Preservation Society (ICRS) [109] cautiously supported PRP, emphasizing standardized protocols. The UK National Institute for Health and Care Excellence (NICE) permits PRP use only under special arrangements requiring informed consent and does not recommend routine use [110]. Orthopedic and sports medicine societies recognize PRP for KOA after the failure of conventional therapy. Many European sports medicine clinics use PRP, highlighting the discrepancies between guidelines and practice. Most insurers classify PRP as investigational and do not cover it, thus impacting its utilization.

However, a conservative treatment approach would require definitive evidence. Guidelines committees demand reproducible, placebo-controlled efficacy data and proof of their structural benefits. Evidence suggests symptomatic relief but lacks a clear disease-modifying effect, showing no halt in OA progression. Without such evidence, PRP remains in between symptom management and disease modification. Guidelines may be changed if large-scale trials show positive outcomes. Academic discourse has continued to evolve. A 2022 *Nature Reviews Rheumatology* commentary noted that the “use of PRP for knee OA is not supported by high-quality RCT results” [63]. However, two years later, several meta-analyses endorsed PRP [63].

There are constantly emerging reports on recommendations for the use of PRP in KOA. Currently, there are changes in the positions of scientific associations compared to the situation described in the systematic review by Phillips et al. [111], who stated that most guidelines remain ambiguous or do not allow formal recommendations regarding PRP to be made, indicating a discrepancy between the latest clinical evidence and official practical recommendations.

Therefore, clinicians should make these decisions. Many orthopedic surgeons offer PRP despite the guidelines, informing patients of their experimental nature and evidence. PRP merits consideration for athletes or military personnel, where maintaining activity is crucial and options are limited because of its safety profile [93]. The literature shows heterogeneity in PRP formulations, dosing protocols, leukocyte content, and outcome measurements, limiting the generalizability of the results. Standardized systems such as PAW and DEPA lack universal adoption, with trials often omitting critical variables such as platelet concentration and activation methods. This review used advanced tools for transparency and credibility. The PRISMA flowchart facilitated reproducibility, whereas the risk of bias assessment identified concerns about randomization and blinding. The GRADE approach enables structured evaluation of evidence certainty, from high to moderate or low, for long-term efficacy. The evidence map shows the study distribution, highlighting leukocyte-poor PRP vs. hyaluronic acid comparisons and revealing underexplored areas. This review’s strengths included the integration of diverse data types, multiple methodological tools, and a comprehensive search strategy.

A strong consensus is forming around the use leukocyte-poor PRP (LP-PRP) formulations. One to three PRP injections are preferred over single or indefinite repeat protocols. No guideline considers PRP disease-modifying yet; only symptomatic improvement has been demonstrated. The VA/DoD guidelines recommend against orthobiologics such as PRP, unless within a research setting.

### 4.10. Limitations and Heterogeneity

Research indicates variability in platelet-rich plasma (PRP) formulations, injection protocols, patient characteristics, and outcome measures. This study’s limitations include the narrative synthesis approach, which prevents the calculation of pooled effect estimates, and the lack of PRP standardization. Publication bias and language restrictions may have led to the exclusion of relevant studies. These discrepancies impede comparisons and underscore the need for standardization in future trials. The current official guidelines do not recommend PRP for KOA, citing insufficient evidence or conflicting results. PRP remains a treatment option to consider when the standard therapies are inadequate. Patients should be informed of existing evidence and their off-label status. As research advances and cost-effectiveness is demonstrated, PRP may receive stronger endorsement. Future research should prioritize large multicenter randomized trials with defined PRP protocols, relevant endpoints, and standardized reporting, including comparative studies on newer injectables and their long-term outcomes. Standardized reporting tools such as the TIDieR and SPIRIT checklists should be considered in future trials to enhance transparency and reproducibility.

### 4.11. Addressing Heterogeneity: Proposed Standards for PRP Preparation and Outcome Measures

To mitigate these issues, future research must adopt standardized reporting guidelines explicitly mandating detailed descriptions of PRP preparation methods, including platelet and leukocyte concentrations, activation methods, injection protocols, and comprehensive follow-up durations. The adoption of guidelines such as PAW (platelet count, activation, and white blood cells) or DEPA (dose, efficiency, purity, and activation) classification systems, along with universally recognized outcome measures (e.g., WOMAC and IKDC), will significantly enhance clarity, reproducibility, and comparability across clinical studies, facilitating stronger evidence-based clinical guidelines.

To address this critical issue, we propose the following standardized approach:

Consensus on PRP Classification:
Clearly categorize PRP based on the following variables:
  - Platelet Concentration: Standardized reporting as the fold increase relative to baseline platelet counts.
  - Leukocyte Content: Explicitly define PRP as leukocyte-rich (LR-PRP) or leukocyte-poor (LP-PRP) with precise numeric thresholds.
  - Activation Method: Consistent reporting of the activation status (activated vs. non-activated) along with activating agents, concentrations, and volumes used.

Standardized Preparation and Injection Protocols:
Studies should implement the following:
  - Validated, commercially available PRP preparation systems approved by regulatory bodies (FDA and EMA) to ensure reproducibility.
  - Uniform injection protocols specifying the number, volume, interval, and delivery method (preferably ultra-sound-guided).

Uniform Outcome Measurement:
  - Adopt core outcome sets to standardize reporting:
  - Pain: VAS/NRS and the WOMAC pain subscale.
  - Function: WOMAC function, IKDC, and KOOS.
  - Imaging: Standardized MRI protocols and cartilage scoring systems.
  - Patient-Reported Outcomes: Patient Global Assessment (PGA) and Quality-of-Life metrics (EQ-5D and SF-36).
  - Adverse Events: Clearly defined and consistently reported.

Enhanced Reporting Guidelines:

Guidelines require adherence to specialized reporting guidelines (e.g., Minimum Information for Studies Evaluating Biologics in Orthopedics—MIBO), similar to PRISMA or CONSORT, to ensure transparency and comprehensive reporting.

Establishment of a Centralized Registry:

Authorities should create an international registry to systematically record detailed PRP preparation methods, patient demographics, injection details, and outcomes, enabling transparent data sharing and meta-analyses.

Regular Expert Consensus Meetings:

International expert panels, including clinicians, researchers, and patient representatives, should convene to periodically refine these standards based on emerging evidence and clinical practice insights.

Implementing these proposed standards will significantly mitigate methodological heterogeneity, enhance the comparability of results, and ultimately strengthen evidence-based recommendations for PRP therapy in knee osteoarthritis management.

### 4.12. Guidance on Patient Selection Criteria for PRP Therapy in KOA

The patient subgroups most likely to benefit include the following:

- Mild-to-moderate KOA: Patients classified as Kellgren–Lawrence grades II–III typically show better outcomes than those with advanced disease.
- Younger age (<60 years): Younger patients tend to have more responsive cartilage and synovial tissue, potentially resulting in greater symptomatic relief.
- Lower BMI (<30 kg/m^2^): Patients with lower body mass indices generally experience more noticeable improvement, possibly due to lower mechanical stress on joints.
- Shorter disease duration: Patients with earlier disease stages or shorter symptom durations (under 5 years) may benefit more significantly.
- Minimal joint deformity and preserved joint mechanics: Patients without substantial varus or valgus malalignment respond more favorably.
- Previous inadequate response to conservative therapies: Patients who have not benefited sufficiently from NSAIDs, exercise, physical therapy, or hyaluronic acid injections might find added relief from PRP.
- Contraindications to steroid or surgical interventions: Patients unable or unwilling to pursue steroid injections or surgical options may particularly benefit from PRP.

The patient subgroups less likely to benefit include the following:

- Advanced KOA (Kellgren–Lawrence grade IV), marked joint space narrowing, severe cartilage loss.
- Significant knee malalignment or instability requiring surgical correction.
- Severe obesity (BMI > 35 kg/m^2^), as high mechanical loading may diminish therapeutic outcomes.
- Using these criteria can help clinicians carefully select patients who are most likely to derive symptomatic benefit from PRP injections, ensuring an optimal balance of patient expectations, treatment effectiveness, and clinical resource use.

## 5. Conclusions

Platelet-rich plasma (PRP) has emerged as a promising therapeutic intervention for KOA by delivering growth factors and cytokines to mitigate inflammation and promote cartilage synthesis. Compared to hyaluronic acid (HA) and corticosteroids, PRP shows consistent benefits and safety as an intra-articular therapy. Future research should prioritize the standardization of PRP preparation protocols and patient stratification. Research indicates that PRP injections provide pain relief and functional improvement lasting 6–12 months, and they show equal or greater efficacy than corticosteroids or HA in trials [37,84]. It has to be noted that some placebo-controlled trials sometimes show no significant difference between PRP and saline at 12 months. Clinicians should interpret these results cautiously. Although some high-quality placebo-controlled trials indicate no significant long-term difference between PRP and saline, the overall clinical evidence suggests that PRP may offer meaningful short-to-medium-term symptomatic relief in certain patient subgroups. PRP’s therapeutic benefit likely involves placebo effects and biological mechanisms, such as inflammation modulation, which could provide transient functional improvements rather than sustained structural changes. Therefore, clinicians should clearly communicate these nuances to patients, emphasizing PRP’s potential symptomatic benefits within a broader multimodal management strategy, rather than positioning it as a definitive long-term, disease-modifying treatment for knee osteoarthritis. Its safety profile is beneficial for patients who cannot tolerate NSAIDs and steroids. However, PRP remains controversial, as placebo-controlled studies have shown no significant difference between PRP and saline. Literature discrepancies reflect varying PRP preparations, and studies should categorize PRP according to platelet count, leukocyte presence, and activation methods. Clinically, PRP is considered for patients seeking alternatives, complementing core OA treatments such as exercise, physical therapy, and weight management. It can be integrated into treatment plans for patients undergoing joint replacement. PRP injections harness the body’s healing ability, with evidence supporting their short-term efficacy and minimal risk. Although not a cure, PRP may improve the quality of life of OA patients. Clinical adoption depends on the standardization and proof of effectiveness, and PRP may serve as an alternative therapy for selecting patients with early to-moderate OA.

## Figures and Tables

**Table 1:** Summary of included studies. The table summarizes the key characteristics and findings of the representative clinical trials and meta-analyses. (One study was excluded because it was a letter to the editor and did not contain information on study design, sample size, treatment protocol, and follow-up duration [25]).

Study	Study Design	Sample Size	Treatment Protocol	Follow-Up Duration	Outcomes
Kon et al., 2011 [23]	Prospective comparative study	150 (PRP: 50 and HA: 100	PRP: 3 injections every 14 days	6 months	Both groups improved, but PRP produced higher IKDC and EQ-VAS scores and a lower re-intervention rate than HA at 6–24 months.
Filardo et al., 2012 [26]	Randomized double-blind prospective trial	109 (PRP: 54 and HA: 55)	3 weekly injections	12 months	PRP and HA both improved pain and function; no significant overall difference at 12 months, although PRP trended better in low-grade OA.
Say et al., 2013 [27]	Prospective, comparative clinical study	90 (PRP: 45 and HA: 45)	PRP: 1 and HA: 3 injections	6 months	Single PRP injection yielded greater reductions in pain (VAS) and WOMAC scores than HA at 6 months.
Khoshbin et al., 2013 [28]	Systematic review	577 (PRP: 264 and Control: 313)	2, 3, or 4 injections	24 weeks	Meta-analysis found PRP reduced WOMAC pain and improved function more than saline or HA at 6 and 12 months without increasing adverse events.
Holguin, 2014 [29]	Prospective study	150 (PRP: 55; HA: 55)	3 weekly injections	12 months	PRP provided significantly greater improvements in WOMAC total and pain vs. HA at 12 months.
Laudy et al., 2014 [30]	Systematic review	1110 (PRP~50% HA ~50%) 10 studies	Three intra-articular injections	6 to 12 months	Systematic review concluded evidence is limited; PRP may offer symptomatic benefit over HA, but heterogeneity prevents firm conclusions.
Raeissadat et al., 2015 [31]	Non-placebo-controlled randomized clinical trial	160 (PRP: 87 and HA: 73)	PRP: 2 injections at 4-week interval and HA: 3 injections at 1-week interval	12 months	At 12 months PRP produced larger decreases in WOMAC pain and stiffness and higher patient satisfaction than HA.
Montañez-Heredia et al., 2015 [32]	Double-blind randomized controlled trial	95	PRP-1: 1, PRP-2: 2, and HA: 3 injections	3 months	No significant difference among single- or double-dose PRP and HA at 3 months; all groups improved similarly.
Filardo et al., 2015 [33]	Randomized double-blind trial	192 (PRP: 94, HA: 89)	3 weekly injections	12 months	Both PRP and HA improved outcomes over 12 months; PRP did not achieve superiority except in younger, less degenerated knees.
Cole et al., 2015 [34]	Double-masked prospective randomized controlled trial	111	3 weekly injections	24 weeks	PRP resulted in lower pain (VAS) and higher IKDC than HA at 24 and 52 weeks; primary WOMAC pain not different.
Lana et al., 2016 [35]	Multi-center, randomized, controlled, double-blind, prospective trial	105 (HA: 36, PRP: 36, and HA + PRP: 33)	3 injections at 2-week intervals	12 months	Combination PRP + HA achieved the greatest reduction in WOMAC pain; PRP alone also outperformed HA at 12 months.
Cunningham, 2017 [36]	Randomized controlled trial	120 (PRP: 60 and HA: 60)	4 weekly injections	24 weeks	PRP produced significantly better WOMAC pain and function scores than HA at 24 weeks.
Dai et al., 2017 [37]	Meta-analysis	1069	Varied among studies	12 months	Meta-analysis: PRP superior to HA for pain and function at 12 months (WOMAC pain MD: −2.83).
Shen et al., 2017 [38]	Systematic review and meta-analysis (17 RCT)	1423 (HA Ozone and Saline)	Varied among studies	12 weeks to 12 months	PRP injections provided greater short-term (≤6 months) pain relief than HA or ozone in pooled RCTs.
Di Martino et al., 2018 [39]	Double-blind randomized controlled trial	192	3 weekly injections	Mean 64.3 months	Early PRP benefit over HA faded; at mean 5-year follow-up, no significant difference in IKDC or KOOS.
Zhang et al., 2018 [40]	Meta-analysis (RCTs)	1524 (PRP: 788 and HA: 736)	PRP 3 and HA 3 (injections once a week)	12 months	Pooled RCTs showed that PRP reduced WOMAC pain and VAS more effectively than HA at 6 and 12 months.
Lin et al., 2019 [41]	Randomized, dose-controlled, placebo-controlled, double-blind, triple-parallel clinical trial	87 knees (53 patients)	3 weekly injections	12 months	Dose-controlled RCT confirmed that PRP improved WOMAC pain and IKDC vs. placebo; benefits sustained for 12 months.
Meheux et al., 2020 [42]	Systematic review	739 patients (817 knees)	No mention found	Up to 12 months	Systematic review found that PRP yielded clinically significant pain and function improvements lasting up to 12 months and exceeded HA in most trials.
Xu et al., 2020 [43]	Prospective cohort study	122 (PRP: 40, HA: 34, and PRP + HA: 48)	PRP: 3 and HA: 3 (injections once a week)	24 months	The PRP + HA combination had greatest WOMAC improvement; PRP alone was better than HA and benefits sustained for up to 24 months.
Wu et al., 2020 [44]	Meta-analysis	1063 (PRP: 526 and HA: 537.	PRP: 3 and HA: 3 (injections once a week)	No mention	Meta-analysis: PRP was superior to HA for VAS and WOMAC pain at 6–12 months, with more mild post-injection pain events.
Li et al., 2020 [45]	Systematic review and meta-analysis	661 (PRP: 338 and A: 323)	Varied among studies	12 months	PRP showed better pain relief and functional scores than HA at 3–12 months in pooled analysis.
Karasavvidis et al., 2020 [46]	Systematic review and meta-analysis	377 (PRP-HA: 193 and HA: 184)	Varied among studies	6 to 12 months	Network meta-analysis indicated that the PRP + HA combination ranked highest for pain reduction, ahead of PRP or HA alone.
Tang et al., 2020 [47]	Meta-analysis (20 RCT)	1281 (PRP: 654 and HA: 627)	Varied among studies (1–4 injections)	3 to 12 months	PRP outperformed HA on VAS and WOMAC across 20 RCTs, with effects persisting for 12 months.
Tan et al., 2020 [48]	Meta-analysis	2430	Varied among studies	12 months	Meta-analysis of 2430 knees showed that PRP provided superior pain relief vs. HA at 6 and 12 months with comparable safety.
Filardo et al., 2020 [49]	Systematic review and meta-analysis	2829 (PRP: 1403 and Control: 1426)	Varied among studies	12 months	Review concluded that PRP offers small-to-moderate clinical benefit over HA, especially in younger patients.
Belk et al., 2023 [50]	Systematic review and meta-analysis	2396 (PRP: 1042, BMAC: 226, and HA: 1128)	Varied among studies	PRP: 13.5 months, BMAC: 17.5 months, and HA: 14.4 months	PRP and BMAC yielded larger mean improvements in WOMAC pain than HA at ~14 months; adverse events similar.
Jivan et al., 2021 [51]	Phase I open-label clinical trial	20 (PRP: 10 and HA: 10)	Varied among studies	12 months	The phase I trial showed a 60% pain reduction with PRP vs. 40% with HA at 12 months; no severe adverse events.
McLarnon and Heron, 2021 [52]	Systematic review and meta-analysis	648	Single or triple injections	12 months	Systematic review: The majority of RCTs favored PRP over HA for WOMAC pain/function at 12 months.
Sdeek et al., 2021 [53]	Prospective, double-blind, randomized controlled trial	189	3 injections every 2 weeks	36 months	PRP maintained significant WOMAC and VAS improvements over HA throughout the 36 months of follow-up.
Singh et al., 2021 [54]	Meta-analysis	PRP, HA, and corticosteroids found	Varied among studies	Minimum 6 months	Network meta-analysis ranked PRP highest for pain relief over HA and corticosteroids at ≥6 months.
Wang et al., 2022 [55]	Prospective, double-blind, parallel, randomized controlled trial	110 (PRP: 54 and HA: 56)	Single injection	6 months	Single-injection RCT found no significant superiority of PRP over HA on WOMAC pain at 6 months.
Branch et al., 2023 [56]	Randomized controlled trial	64	3 injections	24 months	PRP superior to HA for WOMAC at 6 and 12 months, but groups converged by 24 months; safety profiles comparable.
Karas et al., 2023 [57]	Systematic review	No mention found	Varied among studies	12 months	Systematic review affirms consistent PRP advantage over HA across the majority of the included trials.
Li et al., 2023 [58]	Systematic review and meta-analysis	1512	Varied among studies	12 months	Updated meta-analysis showed that PRP offered clinically meaningful reductions in VAS and WOMAC vs. HA at 12 months.
Belk et al., 2023 [50]	Systematic review and meta-analysis	2396 (PRP: 1042, BMAC: 226, and HA: 1128)	Varied among studies	PRP: 13.5 months, BMAC: 17.5 months, and HA: 14.4 months	PRP and BMAC yielded larger mean improvements in WOMAC pain than HA at ~14 months; adverse events similar.
Ivander and Anggono, 2024 [59]	Systematic review	447 (PRP: 198 and HA: 194)	Varied (single and multiple injections)	Varied (1–24 months)	The systematic review found that PRP reduced VAS more than HA at 6 months; combination therapy most effective.
Jawanda et al., 2024 [60]	Systematic review and network meta-analysis	9338 knees	Varied among studies	Minimum 6 months	Network meta-analysis ranked PRP as most efficacious for pain and function at ≥6 months among injectables.
Indra et al., 2024 [61]	Comparative study	No mention found	No mention found	12 months	Comparative study reported significantly greater WOMAC and VAS improvements with PRP over HA at 12 months.

**Table 2:** Summary of PRP treatment effects at 3, 6, and 12 months.

Outcome Measure	3 Months	6 Months	12 Months
Pain reduction (VAS/WOMAC pain)	Significant improvement in most RCTs vs. HA/steroids; some vs. placebo	Sustained benefit in most studies, especially LP-PRP vs. HA	Mixed results; some RCTs show parity with placebo
Functional improvement (WOMAC/IKDC)	Consistent improvement in early to moderate KOA	Functional gains persist in KL I–II; mixed for KL III	Diminishing effect in moderate to severe OA (KL III–IV)
Structural effect (imaging-based)	No structural changes typically detectable	No disease-modifying effect evident	No radiographic progression delay observed
Adverse events	Very few, mostly local, and mild (e.g., injection site pain)	No new safety signals	Safe long-term profiles reported in most studies

**Table 3:** Summary of PRP treatment effects compared to HA at 3, 6, and 12 months.

Outcome Measure	3 Months	6 Months	12 Months
Pain reduction (VAS/WOMAC pain)	Greater reduction than HA in most RCTs, particularly LP-PRP	Sustained superiority over HA; some studies show comparable effects	Mixed findings; several trials show no significant difference from HA
Functional improvement (WOMAC/IKDC)	Significantly better improvement vs. HA, especially in KL I–II	PRP superior to HA in maintaining function in early OA	Functional scores converge in moderate-to-severe OA
Structural effect (imaging-based)	No significant differences vs. HA	No imaging evidence of disease-modifying effects for either	No radiographic progression delay in either group
Adverse events	Similar or lower incidence than HA; mostly mild local reactions	Favorable safety profiles; less post-injection swelling than HA in some studies	Both treatments well tolerated; no major differences

**Table 4:** Summary of functional outcomes comparing PRP and hyaluronic acid (HA) at 3, 6, and 12 months.

Functional Measure	3 Months	6 Months	12 Months
WOMAC Function Score	PRP shows greater improvement than HA in most RCTs; difference typically 10–20 points	Difference maintained; PRP superior in KL I–II; minimal decline in the HA group	Improvements diminish; scores similar in KL III–IV across groups
IKDC Score	PRP-treated patients demonstrate better gains; a 5–10-point advantage vs. HA	Sustained difference in favor of PRP, especially in active individuals	Scores converge; no significant between-group difference in advanced OA
Lequesne Index	PRP significantly reduces the disability score vs. HA	Benefit persists at 6 months for PRP; slight regression in the HA group	Scores align in patients with KL III or higher
KOOS Function Subscale	Marked improvement with PRP vs. HA; a larger effect size in early OA	PRP maintains advantage; HA plateaus or slightly regresses	Minimal between-group difference; PRP slightly better in KL I–II

**Table 5:** Risk of bias assessment for selected key randomized controlled trials included in the review. The domains included randomization, allocation concealment, blinding, the completeness of outcome data, selective reporting, and overall risk, which were evaluated according to Cochrane RoB criteria.

Study	Randomization	Deviations from Interventions	Missing Data	Outcome Measurement	Selective Reporting	Overall Bias
Filardo et al. [26]	Low	Low	Some concerns	Low	Low	Low
Meheux et al. [42]	Some concerns	Low	Low	Low	Low	Some concerns
Patel et al. [69]	Low	Low	Low	Low	Low	Low
Cerza et al. [36]	High	Some concerns	Low	Some concerns	Low	High

**Table 6:** GRADE summary of evidence for PRP in KOA. The GRADE summary table evaluates the certainty of evidence for primary outcomes of PRP in KOA, including pain reduction, function, adverse events, and structural improvement. Certainty was rated across the GRADE domains, such as the risk of bias, inconsistency, and imprecision.

Outcome	No. of Studies	Consistency	Certainty of Evidence (GRADE)	Comments
Pain reduction (PRP vs. HA)	20+ RCTs and six meta-analyses	Moderate to high	Moderate	Consistent benefit at 6–12 months; I^2^ ~ 60–80%
Function improvement (PRP vs. HA)	18+ RCTs and five meta-analyses	Moderate	Moderate	WOMAC and IKDC improvement; some heterogeneity
Pain reduction (PRP vs. corticosteroids)	12+ RCTs and three meta-analyses	Moderate	Low	The short-term effect is similar; the long-term benefit favors PRP
Adverse events (PRP vs. HA or steroids)	10+ RCTs	High	High	Very few serious AEs; mostly mild injection-site pain
Structural improvement (MRI or biomarkers)	5 RCTs and two pilot studies	Low	Low	Exploratory only; insufficient evidence for conclusions

**Table 7:** Summary of clinical guideline recommendations for PRP in KOA.

Organization	Year	Recommendation for PRP	Certainty/Comment
ACR/AF [101]	2019	Not recommended	Limited evidence and high variability
OARSI [102,103]	2019	Uncertain	Heterogeneous studies and bias risk
NICE (UK) [110]	2019	Special arrangements only	Requires governance and consent
GRIP [108]	2020	Recommended as a second-line treatment	Cautious use after other treatments fail
VA/DoD [106]	2020	Not recommended	Insufficient evidence for use
AAOS [104,105]	2021	Inconclusive	Lack of standardized protocols
AOSSM [85]	2022	Promising option	Useful in active patients with KL I–II
ESSKA-ORBIT [107]	2024	Recommended (early OA)	Prefer LP-PRP; 1–3 injections
ESSKA-ICRS [109]	2024	Recommended (post-failure conservative/injective)	Appropriate for KL 0–3, ≤80 years; not a first-line therapy or KL 4
ISAKOS	2025	Observational data	PRP less effective in older patients, males, KL IV, and poor alignment; KL grade strongest predictor

## Data Availability

No new data were obtained for this study.
